# The influence of pure tacts and intraverbals on the transfer of verbal learning to new stimuli: An experimental study in children

**DOI:** 10.3758/s13420-025-00684-1

**Published:** 2025-09-10

**Authors:** Miguel A. Maldonado, Juan Miguel Alcaide, Francisco J. Alós

**Affiliations:** 1 Departamento de Psicología, Facultad de Ciencias de la Educación y Psicología, Universidad de Córdoba, Calle San Alberto Magno, s/n, 14071 Córdoba, España; 2 Hospital Universitario “Reina Sofía”, Instituto Maimónides de Investigación Biomédica de Córdoba (IMIBIC), Córdoba, Spain

**Keywords:** Impure tacts, Verbal behavior, Compound stimuli, Learning transfer, Generative learning, Children, Tactos impuros, Comportamiento verbal, Estímulos compuestos, Transferencia de aprendizaje, Aprendizaje generativo, Niños

## Abstract

This study investigates learning transfer processes in the teaching of pure tacts and intraverbals within the context of verbal behavior. The objectives were: to assess whether training pure tacts and intraverbals, through the inclusion of different stimuli, facilitates learning transfer to new impure tacts, and to determine whether one of these verbal operants (pure tact or intraverbal) better promotes learning transfer. The sample included 54 children aged 11–12 years, using a within-subjects experimental design with pre-post measures. The research was divided into 17 phases and six cycles, with two levels of the independent variable: (1) different stimuli in pure tacts and same in intraverbals, and (2) same stimuli in pure tacts and different in intraverbals. The presentation order was counterbalanced to control for order effects in training. Analyses using repeated-measures ANOVAs revealed that while participants could learn with either type of training, the training of pure tacts facilitated a greater learning transfer toward new impure tacts. In fact, training with pure tacts significantly increased the accuracy of responses in impure tact tests compared to intraverbal training, promoting greater generalization of learning (Pure tacts: M = 80.25%; SD = 4.04; F(1,26) = 5.44, p = .028, ηp^2^ = .173; Intraverbals: M = 51.54%; SD = 3.65; F(1,26) = 8.33, p = .008, ηp^2^ = .243). This finding suggests that pure tacts play a key role in promoting generative behavior, allowing the acquisition of new behavioral repertoires without explicit instruction. Theoretically, this study contributes to isolating the effect of pure tacts in learning transfer, while, practically, it offers relevant educational strategies to improve learning in populations with verbal and cognitive developmental limitations.

**Supplementary Information:**

The online version contains supplementary material available at 10.3758/s13420-025-00684-1.

## Resumen

Este estudio investiga los procesos de transferencia del aprendizaje en la enseñanza de tactos puros e intraverbales en el contexto del comportamiento verbal. Los objetivos fueron: evaluar si el entrenamiento de tactos puros e intraverbales, mediante la inclusión de diferentes estímulos, facilita la transferencia del aprendizaje a nuevos tactos impuros; y determinar si una de estas operantes verbales (tacto puro o intraverbal) promueve mejor la transferencia del aprendizaje. La muestra incluyó a 54 niños de 11 a 12 años, utilizando un diseño experimental intrasujetos con medidas pre y post. La investigación se dividió en 17 fases y 6 ciclos, con dos niveles de la variable independiente: (1) estímulos diferentes en tactos puros e idénticos en intraverbales, y (2) estímulos idénticos en tactos puros y diferentes en intraverbales. El orden de presentación se contrabalanceó para controlar los efectos del orden en el entrenamiento. Los análisis realizados mediante ANOVA de medidas repetidas revelaron que, si bien los participantes podían aprender con cualquier tipo de entrenamiento, el entrenamiento de tactos puros facilitaba una mayor transferencia de aprendizaje hacia nuevos tactos impuros. De hecho, el entrenamiento con tactos puros aumentó significativamente la precisión de la respuesta en las pruebas de tactos impuros en comparación con el entrenamiento intraverbal, lo que promovió una mayor generalización del aprendizaje (tactos puros: M = 80,25 %; DE = 4,04; F (1,26) = 5,44, p = 0,028, ηp^2^ = 0,173; Intraverbal: M = 51,54 %; DE = 3,65; F(1,26) = 8,33, p = 0,008, ηp^2^ = 0,243).Este hallazgo sugiere que los tactos puros desempeñan un papel clave en la promoción del comportamiento generativo, permitiendo la adquisición de nuevos repertorios de comportamiento sin instrucción explícita. En teoría, este estudio contribuye a aislar el efecto de los tactos puros en la transferencia de aprendizaje, mientras que, en la práctica, ofrece estrategias educativas relevantes para mejorar el aprendizaje en poblaciones con limitaciones de desarrollo verbal y cognitivo.

## Introduction

The human capacity to respond to multiple verbal and nonverbal stimuli is fundamental for acquiring new behaviors. Among the various verbal operants proposed by Skinner (1957), pure tacts (PTs) and intraverbals (IVs) play a key role in learning transfer, especially when the stimuli presented varies in complexity and nature. Studying how these operants facilitate learning transfer can have theoretical and applied benefits for understanding complex verbal behavior.

Humans possess the ability, within verbal interactions, to emit responses to physical and/or verbal stimuli, which may also appear in combination. For example, when a young person is reading the greatest novel in the history of Spanish literature, the mother may ask for its title or the author’s name, prompting the young person to respond accordingly: Don Quixote or Miguel de Cervantes. In this example, the youth, by providing an oral verbal response, is attending to two stimuli – the question asked and the book being held. Such scenarios highlight the importance of analyzing how verbal and nonverbal stimuli converge to generate new learning.

In his book Verbal Behavior, Skinner (1957) described a taxonomy which includes tact as “a verbal operant in which a response of a given form is evoked (or at least strengthened) by a particular object or event or property of an object or event” (p. 81-82), and intraverbal as a verbal operant under the control of verbal stimuli, in which there is no point-to-point correspondence with the controlling verbal stimuli. Both operants are maintained by generalized conditioned reinforcement (such as praise or listener attention) (Cooper et al., 2020; Greer & Ross, 2008; Michael, 1982; Rosales et al., 2023; Skinner, 1957).

This theoretical framework accelerated applied and experimental research on verbal behavior (Pérez, 2016; Petursdottir, 2018; Petursdottir & Devine, 2017), although Skinner’s original focus on a single antecedent stimulus does not fully account for more complex verbal interactions (Hayes et al., 2001; Sidman, 1986, 1994; Sivaraman et al., 2023). Hence, more complex forms – like impure tacts – require considering multiple stimuli and their role in learning transfer.

While the triple-contingency paradigm has proven insufficient for explaining more complex interactions, recent research suggests that training verbal operants such as impure tacts may be crucial for understanding flexible verbal responses in novel situations. Similarly, Skinner (1957) described how some phenomena cannot be fully explained through the triple-contingency paradigm, exemplified by “abstract tacts.” Recent works (Guerrero, 2015; Maldonado, 2021; Maldonado et al., 2024) suggest that the inclusion of different stimuli in verbal training may enhance transfer effects. In the present study, such variability was incorporated to increase the contrast between phases, although it was not analyzed as an independent factor.

Recent studies have begun to explore the relationship between tacts and learning transfer in different verbal contexts. For instance, Guerrero (2015) examined the role of combined stimuli in generalizing verbal responses, while Maldonado (2021) demonstrated how impure tacts enable greater flexibility in acquiring new behaviors. However, it remains unclear how variations in stimuli used in pure tacts and intraverbals influence learning transfer. Likewise, Alós et al. (2013) and Guerrero et al. (2015) found that procedures incorporating verbal responses (impure tacts) more effectively promoted new stimulus relations. This underscores the importance of investigating pure tacts and simple intraverbals systematically.

Recent advances in verbal behavior highlight the importance of multiple antecedent stimuli and combinations in determining the complexity of verbal operants (Alós et al., (Alós en prensa); García et al., 2023; Maldonado et al., 2024). For instance, intraverbal research distinguishes between simple intraverbals (one verbal antecedent stimulus) and complex intraverbals (two verbal antecedent stimuli exerting control) (see Belloso-Díaz, 2016; García et al., 2023; Palmer, 2016). Similarly, studies on tacts differentiate between pure tacts (single stimulus) and impure tacts (two stimuli), where pure tacts have a nonverbal or physical antecedent control, and impure tacts have dual control, one verbal and one nonverbal (see Greer & Ross, 2008).

In research on conditional discriminations, various studies have examined the implications of two stimuli on participants’ responses (i.e., to correctly select a name, the listener must pay attention to both stimuli) (see Alonso-Álvarez & Pérez-González, 2006; Debert et al., 2007; Devine et al., 2016; Eikeseth & Smith, 2013; Groskreutz et al., 2010; Guerrero et al., 2020; Lane & Critchfield, 1998; Maguire et al., 1994; Pérez-González & Alonso-Álvarez, 2008; Strommer & Strommer, 1990a, b). Alós et al. (2013) cautioned that a similar phenomenon could occur with impure tacts. The study of two stimuli influencing a response is not new; Axe (2008) and Michael et al. (2011) also examined the necessity of two antecedent stimuli for response emergence (convergent multiple control). However, the number of possible antecedent stimulus and response combinations suggests that both phenomena (compound stimuli and convergent multiple control) are distinct (for more information, see Guerrero, 2015; Maldonado, 2021; Maldonado et al., 2020, 2024).

The implications of tact training (pure and/or impure) on other verbal operants or discriminations have been explicitly investigated in different studies (e.g., Alós et al., 2013; Belloso-Díaz, 2016; Belloso-Díaz & Pérez-González, 2015; Guerrero, 2015; Guerrero et al., 2015; Maldonado, 2021; Maldonado et al., 2020, 2024; Petursdottir et al., 2008). Alós et al. (2013) described a procedure that included compound stimuli in simple discriminations, enabling the precise identification of stimuli and responses in impure tacts. Additionally, Guerrero et al. (2015) observed that procedures incorporating verbal responses (impure tacts), as opposed to selection responses (conditional discriminations), more effectively promoted learning transfer to new stimulus relations, such as conditional discriminations and/or intraverbals.

Recently, Maldonado (2021) and Maldonado et al. (2024) emphasized that impure tacts are not merely the sum of pure tacts and intraverbals, making this form of verbal behavior more complex than the verbal operants initially described by Skinner (1957). For a detailed analysis of complex forms of verbal behavior, readers may consult García et al. (2023). In experiments by Maldonado et al. (2020), (2024) learning transfer to new impure tacts not explicitly taught holds particular importance. This could be considered a form of generative behavior, that is, novel behaviors appearing in new circumstances without direct instruction (see Johnson & Street, 2023). Generativity, combining limited words into novel utterances, underscores the importance of researching impure tacts for both basic and applied purposes (Johnson & Street, 2023; Harley, 2008).

In light of this gap, some studies have begun to address how different stimuli or compound stimuli influence emerging verbal repertoires (e.g., Alós et al., 2013; Guerrero, 2015; Maldonado, 2021). However, the literature still lacks systematic evidence on how stimulus variability interacts with different verbal operants to promote transfer. In this study, such variability was introduced pragmatically to enhance detection of effects, but was not manipulated as a fully crossed experimental variable. This forms the central focus of the present study.

### Objectives and hypotheses

The present study has two main objectives. First, to compare the effectiveness of pure tact (PT) training versus intraverbal (IV) training in promoting the transfer of learning to new impure tacts (ITs). Second, to evaluate whether the order of presentation of the training phases modulates this transfer. Specifically, we hypothesize that training pure tacts first will enhance generalization effects, facilitating subsequent learning in the intraverbal phase, whereas starting with intraverbal training may not produce the same facilitation, or may even reduce it.

Although the use of different and same stimuli was incorporated into the training sequences, this manipulation was not conceptualized as an independent experimental variable, nor was it fully crossed in a factorial design (e.g., no PT-same/IV-same or PT-different/IV-different conditions were implemented). Instead, the introduction of stimulus variability was intended to maximize the contrast between training phases and to increase the sensitivity of the procedure for detecting transfer effects, in line with prior research suggesting that stimulus variability facilitates generalization processes (Guerrero, 2015; Maldonado, 2021, 2024). For this reason, stimulus variability was not included as a factor in the statistical analyses or as part of the specific hypotheses tested. Therefore, the study did not fully cross this factor into a 2 × 2 design, and it was not the focus of specific hypotheses.

The following hypotheses derive from these objectives:

- (H0). No differences are expected in the pretest phase between the participants assigned to each condition, therefore it is expected that they will all show a similar initial performance and that the pretest will confirm the lack of trained responses.
- (H1) Training with pure tacts (PTs) will promote greater transfer of learning to new impure tacts (as evidenced by a higher number of correct trials in post-training transfer tests for impure tacts) than training with intraverbals (IVs).
- (H2) There will be a significant interaction between the type of training and the order of presentation, such that starting with PT will facilitate generalization to IV in the second phase, but starting with IV will not produce the same effect.

## Method

### Design

A 2 × 2 mixed factorial design was used, with a within-subjects factor (type of training: pure tact vs. intraverbal) and a between-subjects factor (order of training: PT→IV vs. IV→PT). The use of same or different stimuli was not manipulated as a separate experimental factor. In addition, pre-test and post-test measurements were introduced to evaluate the evolution of learning transfer.

### Participants

The sample comprised 54 children with typical development from sixth grade, including 25 boys (46.30%) and 29 girls (53.70%), aged between 11 and 12 years (M = 11.5; SD = 0.35), from various public schools in the province of Córdoba, Spain. Public primary schools in Córdoba were selected for participation. The inclusion criteria for participants were:

1. Being enrolled in the sixth year of primary education,
2. Being between 11 and 12 years old,
3. Having a typical developmental level, and
4. Not having any clinical diagnosis.

In addition, participants who did not sign or hand in the informed consent of the guardians or parents to participate were excluded.

Schools were selected by convenience based on accessibility and willingness to participate, provided they met the inclusion criteria. A total of four public primary schools in Córdoba participated in the study with between 12 and 15 participants per school. In each school, participants were randomly selected from the classroom lists, ensuring equal probability of selection, therefore, the sample was probabilistic and randomly selected from a list. A sample size test was carried out using the G power program for a high effect size (0.8) with a confidence level of 0.95% (significance (α) of 0.05), yielding a sample size of 27 participants per group with a statistical power of d = 0.82. Participants were randomly assigned to one of the two training orders using a random number generator in Excel. The first 27 participants were allocated to the PT→IV group (Pure Tact training followed by Intraverbal training) and the remaining 27 to the IV→PT group (Intraverbal training followed by Pure Tact training).

### Setting, materials, and stimuli

Experimental sessions were conducted in a room designated by the educational institution. Each session lasted approximately 45 min per participant. The participant and the researcher sat facing each other, separated by a 1 m-wide table. An opaque screen was placed vertically between them to prevent the participant from observing the researcher’s materials and notes. Additionally, a non-participating observer, seated approximately 2 m behind and to the left of the child, recorded responses for subsequent data reliability. Two types of stimuli were used in the experiment:

- *Nonverbal stimuli*: Cards (12 cm × 18.5 cm) displaying one of four possible images (two boys or two girls).
- *Verbal stimuli*: One of four words presented orally by the researcher: blue, green, red, or yellow.

Participants were required to orally provide one of 12 possible responses. Stimuli and responses were coded with an alphanumeric code consisting of a capital letter (A, B, C, D, F, and G) and a number identifying set x (1 and 2) and set y (3 and 4). This code also included the letter “R” to indicate that it was a response. If the possible responses belonged to different letters, the Greek letter pi (π) was used to identify responses from C and D, and mu (µ) for responses from F and G. Composite stimuli formed by the combination of the card and color name stimuli were identified in the code with parentheses. For example, the alphanumeric code (AxBx)-Rπx includes the following four stimulus relations: (A1B1)-RC1, (A1B2)-RD1, (A2B1)-RC2, (A2B2)-RD2 (see Table 1).

**Table 1:**
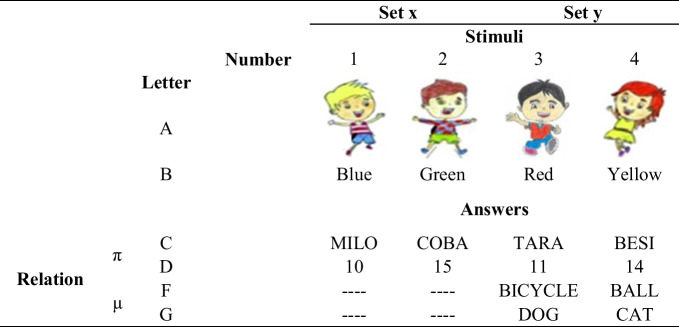
Stimuli and responses used during the experiment

**Fig. 1:**
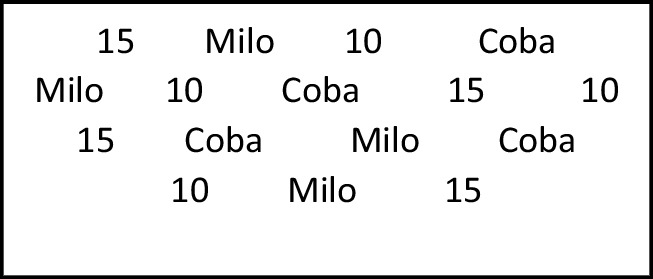
Aid 1: Distribution of possible responses for the child to name

To facilitate the children’s discrimination, the possible responses for each cycle were presented on a printed sheet (a visual aid where terms were arranged horizontally) that included a row with all possible response stimuli for each stimulus set. The responses were randomly printed, and the aid was available during training trials but not in test phases. In total, there were three aid sheets, each used in its corresponding cycle: Aid 1 included the following responses: RC1, RC2, RD1, RD2; Aid 2 included the responses: RF3, RF4, RG3, RG4; and Aid 3 included the responses: RC3, RC4, RD3, RD4. The aid sheet was placed to the right of the participant on a table, 40 cm away (see Fig. 1).

Regarding the instructions and the consequences applied, students were informed that they would participate in an academic activity requiring specific responses when shown cards with images. At the beginning of the activity, each participant received the following instructions: “Hello, it’s great that you’ve come to play with us! The game consists of me showing you, sometimes, pictures of some children, and other times I’ll tell you some colors (blue, green, red, yellow), and you’ll need to say some words or numbers from the sheet on the table (pointing to and saying each of the response options as an example). Try to do your best. Sometimes I won’t be able to tell you if you’re right or wrong, and other times I will, but always do the best you can. Let’s start the game!”

Additionally, before the test phases, it was specified that participants would not be told if their response was correct, but they were encouraged to do their best. However, before the training phases, participants were informed that they would be told if their response was correct or incorrect, with examples given at the beginning. In the training phases, correct responses received social reinforcement with words such as “good,” “great,” “excellent,” etc. When the participant made an error, they were told “no,” and a correction procedure was applied, where the same trial was presented again until the correct response appeared, which was then reinforced. In the test phases, no deliberate consequences were applied.

### Procedure

Firstly, contact was made with state primary schools to request their participation in the study. Written informed consent was obtained from the parents or legal guardians of all participating children. Parents and guardians were thoroughly informed about the study’s purpose, ensuring them that participation was entirely voluntary and that children could withdraw at any time without consequences. Additionally, all collected data were guaranteed to be handled anonymously and confidentially, strictly adhering to personal data protection regulations. This study was conducted following all ethical guidelines outlined in the Declaration of Helsinki, ensuring respect and well-being for participants at all times.

The experimental sessions were conducted in research-equipped classrooms, with each session lasting approximately 45 min per child.

The study followed the same structure for both groups: pre-test → training → post-test. The 54 participants were randomly assigned to one of two presentation orders: PT→IV (training in Pure Tacts followed by Intraverbals) or IV→PT (reverse order).

The procedure included:

- Pre-test: verification of initial responses to impure tacts.
- Training in Pure Tacts.
- Training in Intraverbals.

Post-test: evaluation of learning transferred to impure tacts. The pre-test is applied to verify the absence of initial differences and, after the training, the transfer is evaluated in the post-test phase. The study consisted of 17 phases for PT→IV training and also 17 phases for IV→PT training. Half of the participants did one type of training and the other half did the other type of training. During these phases, visual and verbal stimuli were used in tasks involving pure tacts and intraverbals:

1. Pre-tests for two impure tacts;
2. Teaching of impure tacts;
3. Teaching of pure tacts and intraverbals;
4. Post-test of an impure tact;
5. Teaching of pure tacts and intraverbals; and
6. Post-test of impure tact.

The study included two independent variables:

- IV1: Type of Training Phase (within-subjects factor).

This variable refers to the type of verbal operant trained: Pure Tacts vs. Intraverbals. All participants received both types of training, allowing for a direct comparison of their effectiveness in promoting transfer to new impure tacts.

- IV2: Order of Training (between-subjects factor).

This variable refers to the sequence in which the training phases were presented. Participants were randomly assigned to one of two groups:

- PT→IV group: Pure Tact training followed by Intraverbal training.
- IV→PT group: Intraverbal training followed by Pure Tact training.

Although the introduction of different stimuli in pure tacts and same stimuli in intraverbals (and vice versa) was implemented to maximize the contrast between phases and facilitate transfer detection, this factor was not analyzed as a separate independent variable nor fully crossed into a factorial design (i.e., no PT-same/IV-same or PT-different/IV-different conditions were included). This manipulation was incorporated pragmatically to enhance sensitivity to generative learning effects but was not the focus of specific hypotheses. Each teaching phase was designed to assess participants’ ability to transfer learning to new impure tacts without explicit instruction. Therefore, the dependent variable of the study (DV) was the percentage of correct responses in the impure tact tests conducted after each training phase.

To ensure internal validity and control for potential order effects, the order of training phases (PT→IV vs. IV→PT) was counterbalanced across participants. Each participant completed all 17 phases of the procedure, including both types of training and the corresponding transfer tests. That is, in each teaching phase, participants learned either pure tacts or intraverbals. To maximize the contrast between phases and enhance detection of transfer effects, stimulus variability was introduced pragmatically during the training sequences. Specifically:

- In the PT→IV order, participants were first trained in pure tacts using different stimuli from those used in the impure tact tests, while intraverbals were trained with the same stimuli.
- In the IV→PT order, the sequence was reversed: first intraverbals (with different stimuli), then pure tacts (with same stimuli).

However, stimulus variability was not analyzed as a formal independent variable and was not fully crossed into a factorial design. It was implemented as a procedural strategy to increase contrast between phases and facilitate the observation of transfer effects.

This mixed factorial design allowed us to compare:

- The main effect of training type (Pure Tactics vs. Intraverbals, within participants).
- The effect of training order (PT→IV vs. IV→PT, between participants). Table 2 shows a summary of the variables and hypotheses involved in the study.

**Table 2:** Independent variables, dependent variable and hypotheses tested during the experiment

	Dependent variable	Hypothesis
Independent variable	DV1	
IV1	Type of Verbal Operant Trained (within-subjects) Level 1: Pure Tact Training Level 2: Intraverbal Training	(H0) and (H1)
	Percentage of correct responses in impure tact transfer tests	
IV2	Order of Training (between-subjects) Group 1: PT → IV Training Group 2: IV → PT Training	(H0) and (H2)

The phases of the experiment for both conditions are detailed below.

#### PT→IV training

Training with different stimuli in pure tacts and same stimuli in intraverbals (B_A_) compared to the stimuli used in the training phases for impure tacts (B_C_).

### Impure Tact pre-test phase

#### Phase 1. (AxBx)-Rπx

The experimenter presented a visual stimulus (figure) from set x (A1 or A2) and a color name (green or blue). The participant was expected to respond with one of the following from set x:/Coba/,/Milo/,/ten/or/fifteen/. Twelve trials were presented randomly. The criterion for a correct test performance was ten or more correct trials. Possible stimulus-response combinations were:

(A1B1)-RC1: (A1+Blue)-Milo.
(A1B2)-RD1: (A1+Green)-ten.
(A2B1)-RC2: (A2+Blue)-Coba.
(A2B2)-RD2: (A2+Green)-fifteen.

#### Phase 2. (AyBy)-Rµy

The number of trials and presentation conditions were identical to the previous phase, with only the stimuli (A and B) and required responses changed, all from set y, and the aid sheet replaced. The four stimulus-response combinations were:

(A3B3)-RF3: (A3+Red)-bicycle.
(A3B4)-RG3: (A3+Yellow)-dog.
(A4B3)-RF4: (A4+Red)-ball.
(A4B4)-RG4: (A4+Yellow)-cat.

### Impure Tact training (B_C_)

#### Phase 3. Pure Tact training: Ay-RCy

To facilitate task learning, two aid trials were presented. The experimenter first showed a figure from set y (A3 or A4), pointed to the correct response on the aid sheet, said it aloud, and waited for the child to repeat it, then provided social reinforcement. After these two aids, trials were presented randomly. The criterion for phase change was 12 consecutive correct trials. Possible stimulus-response combinations were:

1. A3-RC3: A3-Tara.
2. A4-RC4: A4-Besi.

#### Phase 4. Pure Tact training: Ay-RDy

This phase was identical to the previous one, except that the required responses differed from the previous phase. The combinations were:

1. A3-RD3: A3-eleven.
2. A4-RD4: A4-fourteen.

#### Phase 5. Impure Tact training: (A3Bx)-Rπ3

Two antecedent stimuli were presented: the figure (A3) and a color name from set x (B1 or B2). The stimulus-response combinations were:

1. (A3-B1)-RC3: A3+blue-Tara.
2. (A3-B2)-RD3: A3+green-eleven.

#### Phase 6. Impure Tact training: (A4Bx)-Rπ4

Two new stimuli were presented, and two new responses were required. Possible stimulus-response combinations were:

(A4B1)-RC4: A4+blue-Besi.
(A4B2)-RD4: A4+green-fourteen.

#### Phase 7. Impure Tact training: (AyBx)-Rπy

This phase was identical to the previous one, except that it included four aid trials. Trials were presented until reaching the criterion. The stimulus-response combinations were those presented in phases 5 and 6.

### Pure Tact training (different stimuli) + Intraverbal training (same stimuli) [PT→IV order]

#### Phase 8. Pure Tact: Ax-RCx

One of two possible stimuli (A1 or A2) was presented, and the child had to say: Milo (C1) or Coba (C2). The number of stimuli, trials, phase change criterion, and presentation conditions were identical to phase 3. Possible stimulus-response combinations were:

1. A1-RC1: A1-Milo.
2. A2-RC2: A2-Coba.

#### Phase 9. Pure Tact training: Ax-RDx

One of two possible stimuli (A1 or A2) was presented, and the child had to say: ten (D1) or fifteen (D2). The number of stimuli, trials, phase change criterion, and presentation conditions were identical to the previous phase. Possible stimulus-response combinations were:

1. A1-RD1: A1-ten.
2. A2-RD2: A2-fifteen.

#### Phase 10. Intraverbals: Bx-Rπ1

This phase was identical to the previous one, except that stimulus B was presented verbally by the experimenter, and the child had to respond orally with one of two alternatives: C1 or D1. Possible stimulus-response combinations were:

1. B1-RC1: blue-Milo.
2. B2-RD1: green-ten.

#### Phase 11. Intraverbals: Bx-Rπ2

This phase was identical to the previous one, except that the required responses were C2 and D2. Possible stimulus-response combinations were:

1. B1-RC2: blue-Coba.
2. B2-RD2: green-fifteen.

### Impure Tact post-test (A_A2_)

#### Phase 12. Test A2: (AxBx)-RXX

This phase was identical to the test in phase 1.

### Pure Tact training (same stimuli) + Intraverbal training (different stimuli) [IV→PT order]

#### Phase 13. Pure Tact training: Ay-RF

Training conditions were similar to those in learning phases. Possible stimulus-response combinations were:

1. A3-RF3: A3-bicycle.
2. A4-RF4: A4-ball.

#### Phase 14. Pure Tact training: Ay-RG

The required responses were G3 and D4. Possible stimulus-response combinations were:

1. A3-RG3: A3-dog.
2. A4-RG4: A4-cat.

#### Phase 15. Intraverbals: By-Rµ3

This phase was identical to the intraverbal training phase. Stimulus B was presented verbally by the experimenter, and the child had to respond orally with one of two alternatives: F3 or G3. Possible stimulus-response combinations were:

1. B3-RC3: red-bicycle.
2. B4-RG3: yellow-dog.

#### Phase 16. Intraverbals: By-Rµ4

This phase was identical to the previous one, except that the required responses were F4 and G4. Possible stimulus-response combinations were:

1. B3-RF4: red-ball.
2. B4-RG4: yellow-cat.

### Impure Tact post-test (A_B2_)

#### Phase 17. Test: (AyBy)-Rµy

This phase was identical to the test in phase 2 (see Table 3).

**Table 3:** Phases of PT→IV training of the experiment, trained and tested verbal operants, consequence application, and number of trials per phase

	Phases	Consequences	Trials	Stimulus relation
Impure Tact pretests (A_A1_ and A_B1_)				
**Impure tact**	*1.* (AxBx)-Rπx	No	12	(A1B1)-RC1; (A1B2)-RD1; (A2B1)-RC2; (A2B2)-RD2
	*2.* (AyBy)-Rµy	No	12	(A3B3)-RF3; (A3B4)-RG3; (A4B3)-RF4; (A4B4)-RG4
**Impure Tacts training (B**_**C**_**)**				
**Pure Tact**	3. Ay-RCy	Yes	12	A3-RC3; A4-RC4
	4. Ay-RDy	Yes	12	A3-RD3; A4-RD4
**Impure tacts**	5. (A3B_X_)-Rπ3	Yes	12	A3B1-RC3; A3B2-RD3
	6. (A4B_X_)-Rπ4	Yes	12	A4B1-RC4; A4B2-RD4
	7. (A_Y_B_X_)-Rπ_Y_	Yes	12	(A3B1)-RC3; (A3B2)-RD3; (A4B1)-RC4; (A4B2)-RD4
**Pure Tact training (different stimuli) + Intraverbal training (same stimuli) [PT→IV order]**				
**Pure Tact**	8. Ax-RCx	Yes	12	A1-RC1; A2-RC2
	9. Ax-RDx	Yes	12	A1-RD1; A2-RD2
**Intraverbal**	10. Bx-Rπ1	Yes	12	B1-RC1; B2-RD1
	11. Bx-Rπ2	Yes	12	B1-RC2; B2-RD2
**. Impure Tact post-test (A**_**A2**_**)**				
**Impure tact**	12. (A_X_B_X_)-RX_X_	No	12	(A1B1)-RC1; (A1B2)-RD1; (A2B1)-RC2; (A2B2)-RD2
**Pure Tact training (same stimuli) + Intraverbal training (different stimuli) [IV→PT order]**				
**Pure Tact**	13. Ay-RF	Yes	12	A3-RF3; A4-RF4
	14. Ay-RG	Yes	12	A3-RG3; A4-RG4
**Intraverbal**	15. By-Rµ3	Yes	12	B3-RF3; B4-RG3
	16. By-Rµ4	Yes	12	B3-RF4; B4-RG4
**Impure Tact post-test (A**_**B2**_**)**				
**Impure tact**	17. (A_Y_B_Y_)-Rµ_Y_	No	12	(A3B3)-RF3; (A3B4)-RF3; (A4B3)-RG4; (A4B4)-RG4

#### IV→PT training

Training with same stimuli in pure tacts and different stimuli in intraverbals (B_A_) compared to the stimuli used in the training phases for impure tacts (B_C_).

In this second variation, the type of training was reordered (BB and BA), counterbalancing the presentation order. Thus, half of the experiment participants were exposed to this variation.

### Observer agreement

The researcher recorded participants’ responses, and an independent observer did the same. Agreement was calculated using the following formula: agreements divided by agreements plus disagreements, multiplied by 100. The observers achieved 98% agreement across all trials in every session with all participants.

### Data analysis

The statistical analyses were structured to evaluate both the main and interaction effects of the experimental manipulations on learning transfer. The primary goals were: (1) to examine whether there were baseline differences between the groups in the pre-test phase, (2) to test the main effect of training type (Pure Tacts vs. Intraverbals), (3) to evaluate the effect of the training order (PT→IV vs. IV→PT), and (4) to explore the interaction between training type and training order. Additionally, the analyses assessed pre- to post-test learning gains within each group.

To verify baseline equivalence, a mixed factorial ANOVA was performed on pre-test data. The within-subjects factor was the type of verbal operant tested (Pure Tacts vs. Intraverbals), and the between-subjects factor was the order of training (PT→IV vs. IV→PT). This analysis confirmed the absence of significant differences between the groups at the outset of the study, thus ensuring the homogeneity of participants’ initial performance levels.

To examine the effects of the interventions in the post-test phase, a 2 × 2 mixed factorial ANOVA was conducted. The within-subjects factor was the type of verbal operant trained (Pure Tacts vs. Intraverbals), and the between-subjects factor was the order of training (PT→IV vs. IV→PT). This analysis allowed for the evaluation of three key effects: first, the main effect of the type of training, to determine whether Pure Tact training produced greater transfer to impure tacts than Intraverbal training; second, the main effect of training order, to assess whether initiating training with Pure Tacts facilitated subsequent learning in the Intraverbal phase; and third, the interaction effect, to explore whether the order of training modulated the transfer effect.

Additionally, within each group, separate repeated-measures ANOVAs were conducted to assess learning gains from pre-test to post-test for each verbal operant. These analyses compared the pre- and post-test performances in Pure Tacts and Intraverbals separately for participants in the PT→IV and IV→PT groups. This approach allowed for the examination of whether each training sequence led to significant improvements in learning transfer.

Effect sizes were reported to complement the statistical significance tests. Partial eta-squared (ηp^2^) was used to estimate the magnitude of the effects in the ANOVAs, and Cohen’s d was calculated for specific comparisons between pre- and post-test scores to quantify the effect size of learning gains. The observed statistical power was also reported to assess the sensitivity of the analyses in detecting the hypothesized effects.

The significance level was set at α = 0.05 for all analyses. Where multiple comparisons were performed, appropriate adjustments, such as Bonferroni correction, were applied to control for Type I error. In cases where the assumption of sphericity was violated, the Huynh-Feldt correction was used to adjust the degrees of freedom in the ANOVAs.

All statistical analyses were performed using IBM SPSS Statistics version 25.0.

## Results

**Fig. 2:**
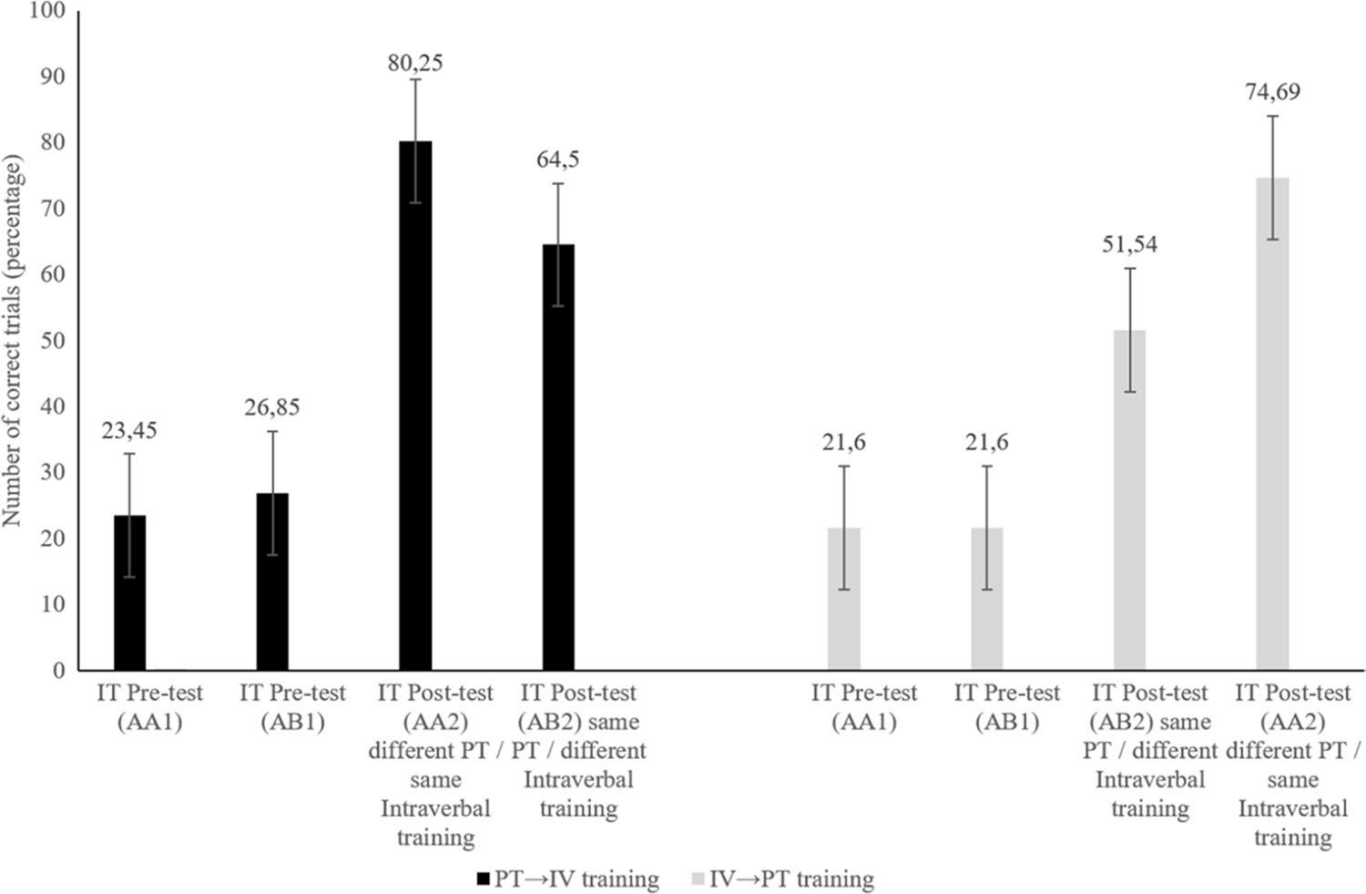
Number of correct trials (in percentages) in the pre-test and post-test of PT→IV training and IV→PT training

Taking into account that a 2 × 2 mixed factorial design was used, with between-subjects factor corresponding to the order of training (PT→IV vs. IV→PT) and a within-subjects factor corresponding to the type of training (Pure Tact vs. Intraverbal), the study evaluated participants’ ability to transfer learning to new impure tacts without explicit instruction. The dependent variable was the percentage of correct responses in the impure tact tests. Figure 2 displays the percentage of correct responses in the impure tact tests, separately for the two training orders: PT→IV (Pure Tact training followed by Intraverbal training) and IV→PT (Intraverbal training followed by Pure Tact training). The error bars in Fig. 2 represent the standard error of the mean, providing information about response variability. As shown, both groups started from similar levels of performance in the pre-test phase, with percentages of correct responses ranging between 21% and 27%. However, after training, performance increased substantially in both groups, as reflected in the post-test results.

Specifically, in the PT→IV group, the post-test of impure tacts after Pure Tact training reached 80%, whereas the post-test of impure tacts after Intraverbal training was around 64%. In contrast, in the IV→PT group, the pattern was reversed: the post-test of impure tacts after Pure Tact training was approximately 52%, while the post-test of impure tacts after Intraverbal training exceeded 74%.

This pattern suggests that, although both groups showed substantial improvements from pre-test to post-test, the relative effectiveness of Pure Tact versus Intraverbal training on transfer to impure tacts varies depending on the order of training. This observation indicates a potential interaction effect, whereby the type of training interacts with the training order to produce differential transfer outcomes.

### Pre-test of impure tacts: Comparison between pure tact and intraverbal training order

To verify that both groups started from equivalent performance levels before the intervention, a mixed factorial ANOVA was conducted on the pre-test data. The within-subjects factor was the type of impure tact test administered (impure tact pre-test before Pure Tact training vs. impure tact pre-test before Intraverbal training), and the between-subjects factor was the training order (PT→IV vs. IV→PT).

Descriptive statistics (n = 27 in each group) showed that in the PT→IV group, the percentage of correct responses in the pre-test was 23.45% (SD = 1.59) for the impure tact test associated with Pure Tact training, and 26.85% (SD = 1.21) for the impure tact test associated with Intraverbal training. In the IV→PT group, the percentages were 21.6% (SD = 1.58) for the Pure Tact pre-test and 21.6% (SD = 1.76) for the Intraverbal pre-test.

The ANOVA results confirmed the absence of statistically significant differences between groups or interaction effects, F(1, 52) = 0.388; p =.536; ηp^2^ =.007; power =.094. This indicates that both groups started from a similar baseline, with no significant differences in the initial performance of impure tact responses. Therefore, any changes observed in the post-test phase can be attributed to the effects of the training interventions rather than pre-existing differences between groups.

### Pre-test versus post-test comparisons: Learning gains within each training order

To analyze whether there were significant improvements from pre-test to post-test within each training order, a repeated-measures ANOVA was conducted including four phases: impure tact pre-test before Pure Tact training, impure tact post-test after Pure Tact training, impure tact pre-test before Intraverbal training, and impure tact post-test after Intraverbal training. This analysis allowed us to assess the learning gains specifically associated with each training sequence.

In the PT→IV group (Pure Tact training followed by Intraverbal training), the ANOVA revealed a significant overall effect, F(3, 78) = 45.906, p <.001, ηp^2^ =.638, power = 1.000, indicating statistically significant differences across the four time points. Post hoc paired comparisons with Bonferroni correction showed substantial improvements from pre-test to post-test in both types of training. For the Pure Tact training, the percentage of correct responses increased from 23.45% (SE = 0.307) in the pre-test to 80.25% (SE = 0.779) in the post-test, with a mean difference of −56.80 percentage points (95% CI [−74.75, −38.85], p <.001). For the Intraverbal training, the scores improved from 26.85% (SE = 0.325) to 64.50% (SE = 0.810), with a mean difference of −37.65 percentage points (95% CI [−54.65, −20.65], p <.001).

In the IV→PT group (Intraverbal training followed by Pure Tact training), the same analysis also showed a significant overall effect, F(3, 78) = 36.492, p <.001, ηp^2^ =.771, power = 1.000. In this group, significant improvements were observed in both cases. For the Pure Tact training, the percentage of correct responses increased from 21.6% (SE = 0.303) in the pre-test to 74.69% (SE = 0.623) in the post-test, with a mean difference of −53.09 percentage points (95% CI [−71.62, −34.56], p =.001). For the Intraverbal training, the improvement was from 21.6% (SE = 0.339) to 51.54% (SE = 0.805), with a mean difference of −29.94 percentage points (95% CI [−49.74, −10.14], p <.001).

These results confirm that, in both training orders, participants showed significant learning gains from pre-test to post-test, reflecting effective transfer of training to new impure tacts. However, the magnitude of improvement varied depending on the type of training and its order, suggesting a potential interaction between these factors.

### Post-test comparisons: Effectiveness of Pure Tact versus Intraverbal training in each group

To evaluate whether the type of training (Pure Tact vs. Intraverbal) produced differential effects in each group during the post-test phase, a repeated-measures ANOVA was conducted separately for each training order. This analysis compared the percentage of correct responses in the impure tact post-tests following Pure Tact training and Intraverbal training.

In the PT→IV group (Pure Tact training followed by Intraverbal training), the analysis revealed a significant effect of the type of training, F(1, 26) = 5.437, p =.028, ηp^2^ =.173, observed power =.612. The mean percentage of correct responses was 80.25% (SD = 4.04) after the Pure Tact training, compared to 64.50% (SD = 3.65) after the Intraverbal training. The difference was 15.75 percentage points (SE = 0.81; 95% CI [1.83, 29.67]; p =.028). These results suggest that, when Pure Tact training is implemented first, it generates higher transfer to impure tacts than Intraverbal training.

In contrast, in the IV→PT group (Intraverbal training followed by Pure Tact training), the pattern was reversed. The repeated-measures ANOVA showed a significant effect of the type of training, F(1, 26) = 8.325, p =.008, ηp^2^ =.243, observed power =.793. The mean percentage of correct responses was 74.69% (SD = 4.18) after the Pure Tact training and 51.54% (SD = 3.23) after the Intraverbal training. The difference was −23.15 percentage points (SE = 0.96; 95% CI [6.43, 39.87]; p =.008). In this case, the Intraverbal training produced higher transfer to impure tacts than the Pure Tact training when administered first.

These findings reveal a cross-over pattern, indicating that the relative effectiveness of Pure Tact versus Intraverbal training depends on the order in which the training is delivered. This observation highlights the presence of an interaction effect between training type and training order in learning transfer.

### Interaction between training type (Pure Tacts vs. Intraverbals) and training order (PT→IV vs. IV→PT)

To analyze the interaction effects between the type of training (Pure Tacts vs. Intraverbals) and the training order (PT→IV vs. IV→PT), a 2 × 2 mixed factorial ANOVA was conducted. The within-subjects factor was the type of verbal operant trained (Pure Tacts vs. Intraverbals), and the between-subjects factor was the order of training (PT→IV vs. IV→PT). The dependent variable was the percentage of correct responses in the impure tact post-tests.

Mauchly’s test of sphericity indicated p ≤.05, with a Greenhouse-Geisser estimate of ε ≥.75. Therefore, the Huynh-Feldt correction was applied to adjust the degrees of freedom in the ANOVA.

### Main effects

The analysis did not reveal a significant main effect of the type of training when averaging across conditions, F(1, 52) = 0.499, p =.483, ηp^2^ =.010, d = 0.20. This indicates that, in general terms, there was no difference in impure tact post-test performance between Pure Tact and Intraverbal training when the order of training was not considered.

Likewise, no significant main effect was found for the training order factor, F(1, 52) = 1.835, p =.181, ηp^2^ =.034, d = 0.38. That is, there were no overall differences in performance between participants who started with Pure Tacts and those who started with Intraverbals when both training sequences were collapsed.

### Interaction effect

However, the analysis revealed a significant interaction between training type and training order, F(1, 52) = 13.756, p =.001, ηp^2^ =.209, d = 1.03, with an observed power of.953. This interaction indicates that the relative effectiveness of Pure Tact versus Intraverbal training on transfer to impure tacts depends on the order in which the training was presented.

**Fig. 3:**
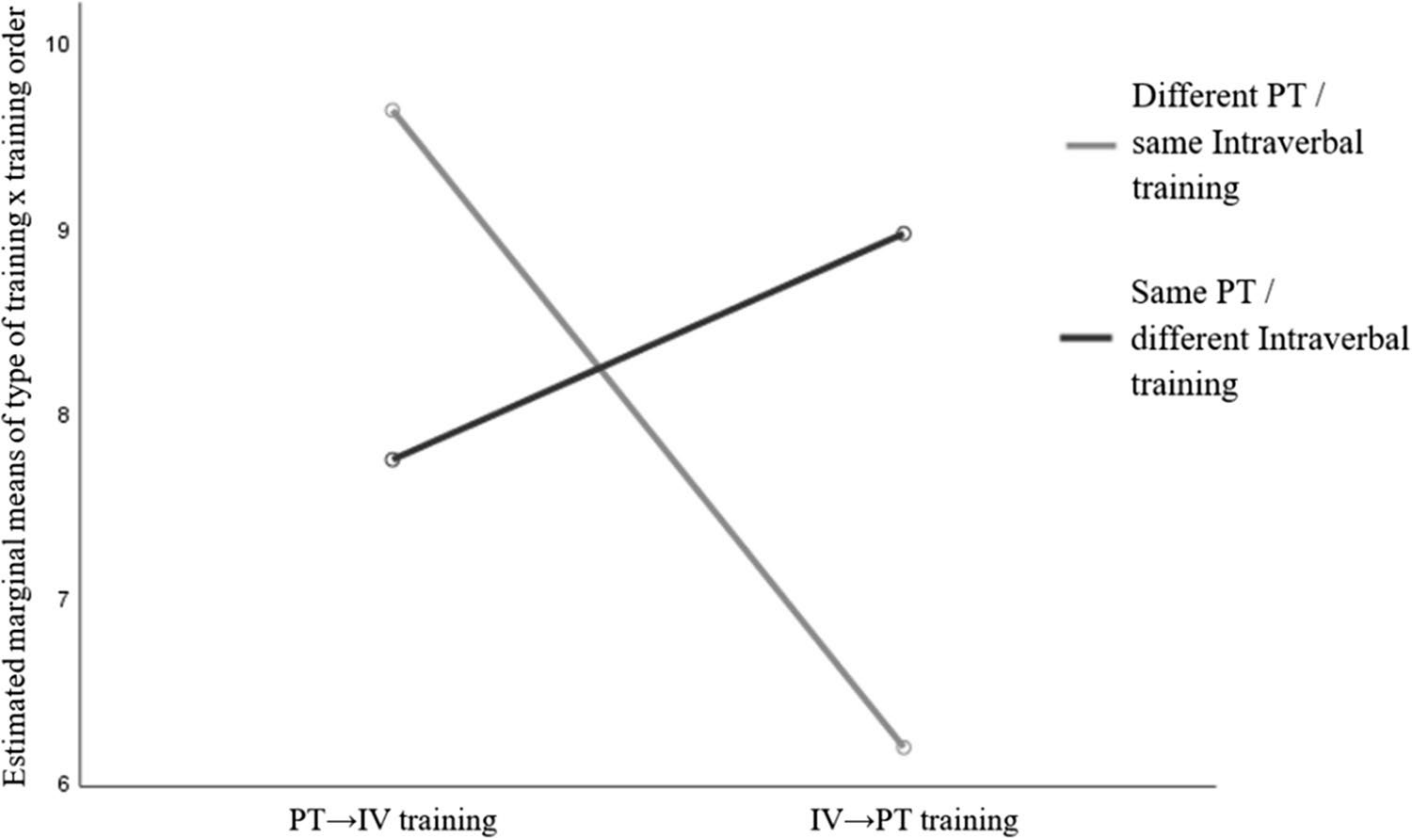
Interaction effect between type of training (Pure Tacts vs. Intraverbals) and training order (PT→IV vs. IV→PT) in the post-test of impure tacts. The graph shows a crossover interaction: In the PT→IV group, Pure Tact training with different stimuli leads to higher transfer than Intraverbal training with the same stimuli. In contrast, in the IV→PT group, Intraverbal training with different stimuli produces better transfer than Pure Tact training with the same stimuli

As shown in Fig. 3, participants in the PT→IV group (who received Pure Tact training first with different stimuli, followed by Intraverbal training with the same stimuli) performed better in the impure tact post-test corresponding to the Pure Tact phase (M = 80.25%, SD = 4.04) compared to the Intraverbal phase (M = 64.50%, SD = 3.65). The effect size for this difference was d = 0.91, indicating a moderate effect.

In contrast, participants in the IV→PT group (who received Intraverbal training first with different stimuli, followed by Pure Tact training with the same stimuli) showed the opposite pattern. Performance in the post-test corresponding to the Intraverbal training was higher (M = 74.69%, SD = 4.18) than in the Pure Tact post-test (M = 51.54%, SD = 3.23), with an effect size of d = 1.13, reflecting a large effect.

This crossover pattern highlights that the benefit of each training type depends on whether the sequence started with Pure Tacts or Intraverbals, supporting the idea that training order and stimulus variability jointly modulate transfer to impure tacts (see Fig. 3).

This figure illustrates the significant interaction between the type of training (Pure Tacts vs. Intraverbals) and the training order (PT→IV vs. IV→PT). In the PT→IV group (Pure Tact training followed by Intraverbal training), the curve corresponding to Different PT/same Intraverbal training reaches higher values, indicating that introducing different stimuli in Pure Tacts and identical stimuli in Intraverbals promotes greater transfer to impure tacts. In contrast, in the IV→PT group (Intraverbal training followed by Pure Tact training), the pattern is reversed: the curve corresponding to Same PT/different Intraverbal training reaches higher values. This crossover interaction suggests that the relative effectiveness of each type of training (Pure Tact or Intraverbal) in promoting transfer depends on the order of training and the associated variability of stimuli. These results are consistent with the statistical interaction observed in the ANOVA. Tables 4 and 5 summarize the detailed ANOVA results, including main effects, simple effects, and the interaction.

**Table 4:** ANOVA summary for each comparison with F(gl), p, ηp2, power, Cohen’s d and means (deviation)

Comparison	ANOVA/Factor(s)	F (gl)	p	ηp^2^	Power	d	Mean (SD)	Interpretation
**Pre-test comparison between Pure Tact and Intraverbal training (both orders)**	Intrasubject factor: Pre-test (Pure Tact vs. Intraverbal pre-test) Intersubject factor: Training order (PT→IV vs. IV→PT)	F(1, 52)= 0.388	0.536	0.007	0.094	0.17	PT→IV: Pure Tact=2.81 (SD=1.59) PT→IV: Intraverbal=3.22 (SD=1.21) IV→PT: Pure Tact=2.59 (SD=1.58) IV→PT: Intraverbal=2.59 (SD=1.76)	No differences or interaction. Both groups started from a similar baseline level.
**Pre- vs. Post-test comparison in PT→IV group**	Repeated measures ANOVA (Pre-test and Post-test for Pure Tact and Intraverbal)	F(3, 78)= 45.906	<.001	0.638	1.000	2.66	Pure Tact: Pre=2.82 (SD=1.59), Post=9.63 (SD=4.04) Intraverbal: Pre=3.22 (SD=1.21), Post=7.74 (SD=3.65)	Significant improvement from pre- to post-test (p<.001) for both Pure Tact and Intraverbal training in PT→IV group.
**Pre- vs. Post-test comparison in IV→PT group**	Repeated measures ANOVA (Pre-test and Post-test for Pure Tact and Intraverbal)	F(3, 78)= 36.492	<.001	0.771	1.000	3.67	Pure Tact: Pre=2.59 (SD=1.58), Post=6.19 (SD=3.23) Intraverbal: Pre=2.59 (SD=1.76), Post=8.96 (SD=4.18)	Significant improvement from pre- to post-test (p<.001) for both Pure Tact and Intraverbal training in IV→PT group.
**Post-test comparison between Pure Tact and Intraverbal training in PT→IV group**	Repeated measures ANOVA (Post-test Pure Tact vs. Intraverbal)	F(1, 26)= 5.437	0.028	0.173	0.612	0.91	Pure Tact: 9.63 (SD=4.04) Intraverbal: 7.74 (SD=3.65)	Pure Tact post-test performance was significantly higher than Intraverbal (p=.028, d=0.91).
**Post-test comparison between Pure Tact and Intraverbal training in IV→PT group**	Repeated measures ANOVA (Post-test Pure Tact vs. Intraverbal)	F(1, 26)= 8.325	0.008	0.243	0.793	1.13	Pure Tact: 6.19 (SD=3.23) Intraverbal: 8.96 (SD=4.18)	Intraverbal post-test performance was significantly higher than Pure Tact (p=.008, d=1.13). Reverse pattern compared to PT→IV.

_*p <.05, **p <.01, ***p <.001_

**Table 5:** Summary of results of the 2 × 2 mixed factorial ANOVA

Comparison	ANOVA/Factor(s)	F (gl)	p	ηp^2^	Power	d	Mean (SD)	Interpretation
**Main effect of Training Type (Pure Tact vs. Intraverbal)**	Intrasubject ANOVA (averaging both orders)	F(1, 52)= 0.499	0.483	0.010	0.095	0.20	Pure Tact=7.20 (SD=3.5) Intraverbal=7.00 (SD=3.3)	No significant differences in overall performance between Pure Tact and Intraverbal training.
**Main effect of Training Order (PT→IV vs. IV→PT)**	Intersubject ANOVA	F(1, 52)= 1.835	0.181	0.034	0.251	0.38	PT→IV=7.40 (SD=3.5) IV→PT=7.20 (SD=3.5)	No significant differences in overall performance between the two training orders.
**Interaction effect (Training Type × Training Order)**	2×2 Mixed Factorial ANOVA	F(1, 52)= 13.756	<.001	0.209	0.953	1.03	–	Significant interaction: The effect of training type depends on the order of training.
**Simple effects in PT→IV group (Post-test comparison)**	Repeated measures ANOVA	F(1, 26)= 5.437	0.028	0.173	0.612	0.91	Pure Tact: 9.63 (SD=4.04) Intraverbal: 7.74 (SD=3.65)	In PT→IV, Pure Tact post-test performance was significantly higher than Intraverbal (p=.028, d=0.91).
**Simple effects in IV→PT group (Post-test comparison)**	Repeated measures ANOVA	F(1, 26)= 8.325	0.008	0.243	0.793	1.13	Pure Tact: 6.19 (SD=3.23) Intraverbal: 8.96 (SD=4.18)	In IV→PT, Intraverbal post-test performance was significantly higher than Pure Tact (p=.008, d=1.13).

_*p <.05, **p <.01, ***p <.001_

In addition, Table 5 presents a summary of the results of the 2 × 2 Mixed Factorial ANOVA with the main effects, the simple effects and the interaction.

Regarding the main effects, no statistically significant differences were observed in the within-subjects factor (type of training: Pure Tacts vs. Intraverbals), *F*(1, 52) = 0.499, *p* = 0.483, ηp^2^ = 0.010, *d* = 0.20, indicating that, when averaging across conditions, the type of verbal operant trained did not produce differential performance in the impure tact tests. Similarly, no significant differences were found in the between-subjects factor (training order: PT→IV vs. IV→PT), *F*(1, 52) = 1.835, *p* = 0.181, ηp^2^ = 0.034, *d* = 0.38, suggesting that, on average, the order of training did not directly affect overall performance.

However, the analysis revealed a significant interaction effect between the type of training and the training order, *F*(1, 52) = 13.756, *p* = 0.001, ηp^2^ = 0.209, *d* = 1.03, with an observed power of 0.953. This interaction indicates that the relative effectiveness of Pure Tact versus Intraverbal training on the transfer to impure tacts depended on whether the training sequence started with Pure Tacts or Intraverbals.

Simple effects analyses clarified this pattern. In the group that received Pure Tact training first (PT→IV training), participants obtained higher post-test scores after Pure Tact training (M = 9.63, SD = 4.04) than after Intraverbal training (M = 7.74, SD = 3.65), with a moderate effect size (*d* = 0.91). Conversely, in the group that started with Intraverbal training (IV→PT training), the pattern was reversed: participants performed better in the post-test after Intraverbal training (M = 8.96, SD = 4.18) than after Pure Tact training (M = 6.19, SD = 3.23), with a large effect size (*d* = 1.13).

These results highlight that the impact of the type of training on learning transfer is not uniform but is modulated by the order in which the training phases are presented. Specifically, training that combines different stimuli in Pure Tacts and identical stimuli in Intraverbals leads to better transfer in the PT→IV sequence, while the reverse pattern is observed when the training starts with Intraverbals.

### Magnitude and power observed

The analyses revealed large effect sizes in the pre-test versus post-test comparisons, indicating robust learning gains following the intervention. In the PT→IV training group, the partial eta squared (ηp^2^) was 0.638, and in the IV→PT training group it reached 0.771. These values correspond to large effects, suggesting that both training sequences led to substantial improvements in performance, with statistical power close to 1.000.

When comparing the two post-tests within each group (Pure Tacts vs. Intraverbals after training), the effect sizes were also meaningful but varied depending on the training sequence. In the PT→IV group, the partial eta squared was 0.173, reflecting a moderate effect size. In the IV→PT group, ηp^2^ was 0.243, indicating a large effect size for the comparison between training types within this group.

In the overall 2 × 2 mixed factorial ANOVA, the interaction between type of training and training order was statistically significant, with ηp^2^ = 0.209, meaning that approximately 21% of the variance in the transfer to impure tacts was explained by the combination of both factors. This represents a moderate-to-large interaction effect. The observed statistical power was high across analyses (e.g., power = 0.953 for the interaction), minimizing the likelihood of Type II error and supporting the reliability of the findings.

Regarding the magnitude of the differences, the estimated Cohen’s *d* for the pre-test vs. post-test comparisons indicated very large effects (d = 2.66 in the PT→IV group and d = 3.67 in the IV→PT group). For the comparison between the two post-tests within each group (Pure Tacts vs. Intraverbals), the effect sizes were also large, with d = 0.91 in the PT→IV group and d = 1.13 in the IV→PT group. The opposite directions of these effects between groups explain the crossover interaction observed in the analysis (global interaction effect: d = 1.03).

Taken together, these parameters confirm that the intervention produced significant learning gains in both training orders. However, the relative effectiveness of Pure Tact versus Intraverbal training on transfer to new impure tacts varied depending on whether participants first received Pure Tact or Intraverbal instruction.

Performance data for all participants, disaggregated by phase, can be found in Tables A1 and A2 in Appendix 1.

## Discussion

The present study aimed to examine how the type of training – pure tact (PT) versus intraverbal (IV) – and the order of presentation of training phases influence the transfer of learning to new impure tacts (IT). Specifically, we hypothesized that training with pure tacts first would facilitate greater generalization, enhancing performance in subsequent intraverbal tasks, whereas starting with intraverbal training might not produce the same facilitation. The results support these objectives and confirm the proposed hypotheses. Participants in both groups started from equivalent baseline levels in the pre-tests, confirming the absence of initial differences and ensuring that the observed improvements in the post-test phase are attributable to the intervention.

The findings of the present study revealed that the order of training plays a pivotal role in modulating learning transfer to new impure tacts. Specifically, when participants received pure tact (PT) training first, with different stimuli, their subsequent performance in the intraverbal (IV) phase showed a notable facilitation of generalization, with higher percentages of correct responses and larger effect sizes. This pattern indicates that starting with pure tacts, especially when incorporating stimulus variability, establishes a broader and more flexible verbal repertoire that can be more easily transferred to new relational contexts, such as those involved in intraverbal responding.

In contrast, when participants began training with intraverbals (IV→PT sequence), the same facilitation effect was not observed. In fact, the data suggest that initiating training with intraverbals may reduce the transfer potential to new pure tacts, possibly because intraverbal learning relies on specific stimulus-stimulus relations that do not necessarily promote generalized responding. This is consistent with prior research suggesting that intraverbal repertoires are less prone to spontaneous generalization, as they tend to involve more rigid stimulus control (Petursdottir & Devine, 2017; Sundberg, 2015).

These results support the idea that stimulus variability, when combined with an initial focus on pure tact training, enhances learning flexibility and generalization (Guerrero, 2015; Maldonado et al., 2024). Teaching pure tacts with varied stimuli may foster the formation of broader stimulus classes and promote multiple exemplar learning, facilitating the emergence of novel relational responses without direct training (Greer & Ross, 2008). In other words, this strategy may strengthen the functional transfer between verbal operants, allowing learners to respond adaptively in new situations.

This pattern of results provides empirical evidence for a crossover interaction, where the relative effectiveness of each training type depends on the order in which it is presented. Therefore, the findings emphasize the importance of sequencing teaching phases strategically, rather than treating verbal operant instruction as isolated units. These insights have both theoretical implications – contributing to models of verbal generativity (Johnson & Street, 2023) – and practical relevance for designing interventions that aim to promote flexible and generalized verbal behavior. These findings have several theoretical implications. First, they reinforce the idea that stimulus variability is a fundamental mechanism for promoting generalization in verbal learning, consistent with previous studies (Guerrero, 2015; Maldonado et al., 2024; Pérez, 2016; Petursdottir, 2018). The significant interaction observed in this study suggests that verbal operants do not function uniformly, but rather that their effectiveness in facilitating transfer depends on how they are taught and on the variability of the stimuli involved. This expands the conceptual framework proposed by Skinner (1957), whose analysis of verbal behavior highlighted the role of operants such as tacts and intraverbals, but did not explicitly address the role of stimulus variability or the sequential effects of training order.

Subsequent research has emphasized the importance of compound stimuli, multiple control, and relational framing in promoting flexible and generative verbal repertoires (Greer & Ross, 2008; Hayes et al., 2001; Johnson & Street, 2023; Sidman, 1986). The present study contributes to this line of research by providing empirical evidence that the combination of stimulus diversity with an initial focus on pure tact training fosters greater transfer and generalization, likely because it facilitates the formation of broad stimulus classes and enhances the learner’s capacity to respond to novel verbal contexts. These results are consistent with recent theoretical perspectives on verbal behavior, emphasizing that transfer is not merely a function of repetition or direct teaching but involves the capacity to integrate multiple sources of control and respond flexibly to new contexts – a process known as verbal generativity (Johnson & Street, 2023; Sundberg, 2015). This aligns with research on composite stimuli and multiple control mechanisms in the development of complex verbal repertoires (García et al., 2023; Maldonado et al., 2024). This study provides a novel contribution to the theory of verbal behavior by highlighting the role of stimulus variability in enhancing the flexibility and adaptability of verbal repertoires. Variability in training contexts appears to facilitate the generalization of learned responses to novel situations, supporting the development of more generative and transferable verbal behaviors (Pérez, 2016; Petursdottir, 2018). From an applied perspective, these findings suggest that educational and therapeutic interventions should prioritize training with diverse stimuli to promote generalization. This approach may be particularly beneficial for individuals with difficulties in verbal development, such as those with autism spectrum disorder, where promoting transfer of learning is a critical challenge (Degli Espinosa et al., 2021; Ismail & Baker, 2023; Vascelli et al., 2024). Adapting training sequences to individual learner profiles and incorporating variability in antecedent stimuli may optimize intervention outcomes in naturalistic settings (Cooper et al., 2020; Greer & Ross, 2008; Petursdottir & Devine, 2017).

## Limitations and future lines of research

Despite the robustness of the results, certain limitations should be considered. First, although the sample size (27 participants per group) was adequate according to the power analysis, this amount could limit the generalization of the findings to broader or more heterogeneous populations. Future research should include larger and more diverse samples, covering different levels of verbal and cognitive development.

Another limitation lies in the controlled experimental context in which the study was carried out. Although this environment allows for greater precision in the measurement of variables, it may not fully reflect the natural conditions of learning in educational or clinical settings. Therefore, it is essential that future studies adopt an ecological approach, integrating interventions into real contexts (e.g., school classrooms) to evaluate the transfer of learning in everyday situations.

Finally, the study focused only on two verbal operants: pure tacts and intraverbals. Given that other operants (such as mands or echoics) are also essential for language development, future research could broaden the scope of the study and include these operants to gain a more comprehensive understanding of the mechanisms that facilitate generalization and verbal generativity (Greer et al., 2017; Sundberg, 2015).

Additionally, future research could further explore how training with the same versus different stimuli modulates generalization processes in impure tacts. Although stimulus variability was incorporated in this study, a more systematic manipulation of this factor in isolation could clarify its specific contribution to verbal learning transfer.

## Conclusion

In summary, this study demonstrates that the order in which verbal operants are taught plays a critical role in facilitating transfer to impure tacts, particularly when pure tact training with varied stimuli precedes intraverbal training. The findings highlight that neither the type of training nor stimulus variability alone fully explain transfer effects, but rather the interaction between these factors is essential. Starting with pure tacts under conditions of stimulus variability appears to enhance generalization, supporting a more flexible and generative verbal repertoire.

These results offer both theoretical and applied contributions. They extend current models of verbal behavior by illustrating how training sequences and stimulus manipulation can modulate verbal learning transfer. From an applied perspective, the findings suggest that educational and therapeutic programs should strategically incorporate varied stimuli and consider the sequencing of teaching phases to maximize generalization.

Although the study presents limitations related to sample size and the controlled experimental setting, it provides a foundation for future research aimed at integrating these procedures into real-world contexts and expanding the investigation to other verbal operants. This line of inquiry may contribute to more effective strategies for promoting verbal generativity and adaptive communication behaviors.

## Supplementary Information

Below is the link to the electronic supplementary material.

Supplementary file1 (DOCX 46 KB)

Supplementary file2 (DOCX 47 KB)

## Data Availability

All data generated or analyzed during this study are included in this published article.
