# Supplementary material for: The influence of pure tacts and intraverbals on the transfer of verbal learning to new stimuli: An experimental study in children
Source: Learn Behav. 2025 Sep 10;54(2):187–203. doi: 10.3758/s13420-025-00684-1 (PMC13194207; doi:10.3758/s13420-025-00684-1)
Supplement: Supplementary file 1 — Supplementary file1 (DOCX 46 KB) [file 13420_2025_684_MOESM1_ESM.docx]

**Appendix 1**

**Table A1**

*Phases of PT→IV training of the experiment, trained and tested verbal operants, consequence application, and number of trials per phase*

| **Participants** | | | | | | | | | | | | | | | | | | | | | | | | | | | | | | |
| --- | --- | --- | --- | --- | --- | --- | --- | --- | --- | --- | --- | --- | --- | --- | --- | --- | --- | --- | --- | --- | --- | --- | --- | --- | --- | --- | --- | --- | --- | --- |
|  | **Phases** | **Consequences** | **Trials** | **1** | **2** | **3** | **4** | **5** | **6** | **7** | **8** | **9** | **10** | **11** | **12** | **13** | **14** | **15** | **16** | **17** | **18** | **19** | **20** | **21** | **22** | **23** | **24** | **25** | **26** | **27** |
|  |  |  |  | **♂** | **♀** | **♂** | **♀** | **♀** | **♂** | **♀** | **♂** | **♂** | **♂** | **♂** | **♀** | **♀** | **♂** | **♀** | **♂** | **♀** | **♀** | **♀** | **♂** | **♂** | **♀** | **♂** | **♀** | **♀** | **♂** | **♀** |
| **Cycle 1. IT Pre-tests (A_A1_ y A_B1_)** | | | | | | | | | | | | | | | | | | | | | | | | | | | | | | |
| **Impure Tact** | 1. | No | 12 | 2 | 6 | 4 | 3 | 4 | 3 | 1 | 3 | 3 | 3 | 5 | 4 | 1 | 0 | 2 | 6 | 0 | 2 | 4 | 2 | 4 | 3 | 3 | 1 | 2 | 1 | 4 |
| **Impure Tact** | 2. | No | 12 | 2 | 3 | 4 | 5 | 2 | 5 | 4 | 4 | 6 | 2 | 5 | 3 | 4 | 4 | 2 | 3 | 4 | 3 | 1 | 4 | 3 | 2 | 2 | 2 | 2 | 3 | 3 |
| **Cycle 2. IT Training (B_C_)** | | | | | | | | | | | | | | | | | | | | | | | | | | | | | | |
| **Pure Tact** | 3. | Yes | 12 | 12 | 12 | 12 | 12 | 12 | 12 | 12 | 12 | 12 | 12 | 12 | 12 | 12 | 12 | 12 | 12 | 12 | 12 | 12 | 12 | 12 | 12 | 12 | 12 | 12 | 12 | 12 |
| **Pure Tact** | 4. | Yes | 12 | 12 | 12 | 12 | 12 | 12 | 12 | 12 | 12 | 12 | 13 | 12 | 12 | 12 | 12 | 12 | 12 | 12 | 12 | 12 | 12 | 12 | 12 | 12 | 12 | 12 | 12 | 12 |
| **Impure Tact** | 5. | Yes | 12 | 12 | 12 | 12 | 12 | 12 | 12 | 12 | 14 | 12 | 12 | 12 | 13 | 12 | 12 | 12 | 12 | 12 | 12 | 12 | 12 | 12 | 12 | 12 | 12 | 12 | 12 | 12 |
| **Impure Tact** | 6. | Yes | 12 | 20 | 12 | 12 | 12 | 12 | 12 | 12 | 12 | 12 | 13 | 12 | 12 | 12 | 12 | 12 | 12 | 12 | 12 | 12 | 12 | 12 | 12 | 12 | 12 | 12 | 12 | 12 |
| **Impure Tact** | 7. | Yes | 12 | 12 | 14 | 14 | 13 | 12 | 28 | 12 | 22 | 15 | 12 | 23 | 12 | 14 | 12 | 12 | 20 | 12 | 12 | 21 | 12 | 12 | 12 | 12 | 12 | 12 | 12 | 12 |
| **Cycle 3. PT + I (B_A_) Training** | | | | | | | | | | | | | | | | | | | | | | | | | | | | | | |
| **Pure Tact** | 8. | Yes | 12 | 12 | 12 | 12 | 12 | 12 | 12 | 12 | 12 | 12 | 13 | 12 | 12 | 12 | 12 | 12 | 12 | 12 | 12 | 12 | 12 | 12 | 12 | 12 | 12 | 12 | 12 | 12 |
| **Pure Tact** | 9. | Yes | 12 | 12 | 12 | 12 | 12 | 12 | 12 | 12 | 12 | 13 | 12 | 12 | 12 | 12 | 12 | 12 | 12 | 12 | 12 | 12 | 12 | 12 | 12 | 12 | 12 | 12 | 12 | 12 |
| **Intraverbal** | 10. | Yes | 12 | 12 | 12 | 12 | 12 | 12 | 12 | 12 | 12 | 12 | 12 | 12 | 12 | 12 | 12 | 12 | 12 | 12 | 12 | 12 | 12 | 12 | 12 | 12 | 12 | 12 | 12 | 12 |
| **Intraberval** | 11. | Yes | 12 | 12 | 12 | 12 | 12 | 12 | 12 | 13 | 12 | 12 | 12 | 12 | 12 | 12 | 12 | 12 | 12 | 12 | 12 | 12 | 12 | 12 | 12 | 12 | 12 | 12 | 12 | 12 |
| **Cycle 4. IT Post-test (A_A2_)** | | | | | | | | | | | | | | | | | | | | | | | | | | | | | | |
| **Impure Tact** | 12. | No | 12 | 3 | 11 | 12 | 11 | 12 | 12 | 2 | 12 | 12 | 12 | 5 | 7 | 12 | 2 | 2 | 12 | 12 | 1 | 12 | 12 | 12 | 12 | 12 | 12 | 12 | 12 | 12 |
| **Cycle 5. PT + I (B_B_) Training** | | | | | | | | | | | | | | | | | | | | | | | | | | | | | | |
| **Pure Tact** | 13. | Yes | 12 | 12 | 12 | 12 | 12 | 12 | 12 | 12 | 12 | 12 | 12 | 12 | 12 | 12 | 12 | 12 | 12 | 12 | 12 | 12 | 12 | 12 | 12 | 12 | 12 | 12 | 12 | 12 |
| **Pure Tact** | 14. | Yes | 12 | 12 | 12 | 12 | 12 | 12 | 12 | 12 | 12 | 12 | 12 | 12 | 12 | 12 | 12 | 12 | 12 | 12 | 12 | 12 | 12 | 12 | 12 | 12 | 12 | 12 | 12 | 12 |
| **Intraberval** | 15. | Yes | 12 | 12 | 12 | 13 | 12 | 12 | 12 | 12 | 12 | 12 | 12 | 12 | 12 | 12 | 12 | 12 | 12 | 12 | 12 | 12 | 12 | 12 | 12 | 12 | 12 | 12 | 12 | 12 |
| **Intraberval** | 16. | Yes | 12 | 16 | 12 | 13 | 12 | 12 | 12 | 12 | 12 | 12 | 12 | 12 | 12 | 12 | 12 | 12 | 12 | 12 | 12 | 12 | 12 | 12 | 12 | 12 | 12 | 12 | 12 | 12 |
| **Cycle 6. IT Post-test (A_B2_)** | | | | | | | | | | | | | | | | | | | | | | | | | | | | | | |
| **Impure Tact** | 17. | No | 12 | 5 | 6 | 11 | 10 | 12 | 7 | 3 | 12 | 10 | 12 | 12 | 6 | 4 | 3 | 6 | 12 | 12 | 4 | 4 | 4 | 5 | 12 | 12 | 5 | 5 | 3 | 12 |
