# Supplementary material for: The influence of pure tacts and intraverbals on the transfer of verbal learning to new stimuli: An experimental study in children
Source: Learn Behav. 2025 Sep 10;54(2):187–203. doi: 10.3758/s13420-025-00684-1 (PMC13194207; doi:10.3758/s13420-025-00684-1)
Supplement: Supplementary file 2 — Supplementary file2 (DOCX 47 KB) [file 13420_2025_684_MOESM2_ESM.docx]

**Appendix 2**

**Table A2**

*Phases of IV→PT training of the experiment, trained and tested verbal operants, consequence application, and number of trials per phase*

| **Participants** | | | | | | | | | | | | | | | | | | | | | | | | | | | | | | |
| --- | --- | --- | --- | --- | --- | --- | --- | --- | --- | --- | --- | --- | --- | --- | --- | --- | --- | --- | --- | --- | --- | --- | --- | --- | --- | --- | --- | --- | --- | --- |
|  | **Phases** | **Consequences** | **Trials** | **28** | **29** | **30** | **31** | **32** | **33** | **34** | **35** | **36** | **37** | **38** | **39** | **40** | **41** | **42** | **43** | **44** | **45** | **46** | **47** | **48** | **49** | **50** | **51** | **52** | **53** | **54** |
|  |  |  |  | **♀** | **♂** | **♀** | **♂** | **♀** | **♀** | **♂** | **♀** | **♂** | **♂** | **♀** | **♂** | **♀** | **♂** | **♀** | **♀** | **♂** | **♂** | **♀** | **♀** | **♂** | **♀** | **♀** | **♂** | **♀** | **♀** | **♂** |
| **Cycle 1. IT Pre-tests (A_A1_ y A_B1_)** | | | | | | | | | | | | | | | | | | | | | | | | | | | | | | |
| **Impure Tact** | 1. | No | 12 | 0 | 3 | 1 | 6 | 4 | 5 | 3 | 2 | 4 | 3 | 0 | 1 | 3 | 2 | 3 | 1 | 2 | 5 | 2 | 5 | 3 | 3 | 0 | 2 | 2 | 3 | 2 |
| **Impure Tact** | 2. | No | 12 | 3 | 3 | 5 | 1 | 0 | 4 | 1 | 5 | 1 | 7 | 5 | 3 | 1 | 3 | 4 | 3 | 1 | 1 | 2 | 2 | 4 | 3 | 3 | 3 | 0 | 2 | 0 |
| **Cycle 2. IT (B_C_) Training** | | | | | | | | | | | | | | | | | | | | | | | | | | | | | | |
| **Pure Tact** | 3. | Yes | 12 | 12 | 12 | 12 | 12 | 12 | 12 | 13 | 12 | 12 | 12 | 12 | 12 | 12 | 12 | 12 | 12 | 12 | 12 | 12 | 12 | 12 | 12 | 12 | 12 | 12 | 12 | 12 |
| **Pure Tact** | 4. | Yes | 12 | 12 | 12 | 12 | 12 | 12 | 12 | 12 | 12 | 12 | 12 | 12 | 12 | 13 | 13 | 12 | 12 | 12 | 12 | 12 | 12 | 12 | 12 | 12 | 12 | 12 | 12 | 12 |
| **Impure Tact** | 5. | Yes | 12 | 12 | 12 | 12 | 12 | 12 | 12 | 12 | 12 | 12 | 14 | 12 | 12 | 12 | 12 | 12 | 12 | 12 | 12 | 12 | 12 | 12 | 12 | 12 | 12 | 12 | 12 | 12 |
| **Impure Tact** | 6. | Yes | 12 | 12 | 12 | 12 | 12 | 12 | 13 | 12 | 12 | 12 | 13 | 12 | 12 | 13 | 12 | 12 | 12 | 12 | 12 | 12 | 12 | 12 | 12 | 12 | 12 | 12 | 12 | 12 |
| **Impure Tact** | 7. | Yes | 12 | 12 | 23 | 12 | 16 | 12 | 12 | 16 | 19 | 12 | 12 | 12 | 12 | 14 | 12 | 20 | 12 | 12 | 12 | 12 | 12 | 12 | 12 | 16 | 17 | 12 | 12 | 12 |
| **Cycle 3. PT + I (B_B:_) Training** | | | | | | | | | | | | | | | | | | | | | | | | | | | | | | |
| **Pure Tact** | 8. | Yes | 12 | 12 | 12 | 12 | 12 | 12 | 12 | 12 | 12 | 12 | 12 | 12 | 12 | 13 | 12 | 12 | 12 | 12 | 12 | 12 | 12 | 12 | 12 | 12 | 12 | 12 | 12 | 12 |
| **Pure Tact** | 9. | Yes | 12 | 12 | 12 | 12 | 12 | 12 | 12 | 12 | 12 | 12 | 12 | 12 | 12 | 12 | 12 | 12 | 12 | 12 | 12 | 12 | 12 | 12 | 12 | 12 | 12 | 12 | 12 | 12 |
| **Intraverbal** | 10. | Yes | 12 | 12 | 12 | 12 | 12 | 12 | 12 | 12 | 12 | 12 | 12 | 12 | 12 | 12 | 12 | 12 | 12 | 12 | 12 | 12 | 12 | 12 | 12 | 12 | 12 | 12 | 12 | 12 |
| **Intraberval** | 11. | Yes | 12 | 12 | 12 | 12 | 12 | 12 | 12 | 13 | 12 | 12 | 12 | 12 | 12 | 12 | 12 | 12 | 12 | 12 | 12 | 12 | 12 | 12 | 12 | 12 | 12 | 12 | 12 | 12 |
| **Cycle 4. IT Post-test (A_A2_)** | | | | | | | | | | | | | | | | | | | | | | | | | | | | | | |
| **Impure Tact** | 12. | No | 12 | 8 | 5 | 7 | 2 | 11 | 5 | 5 | 5 | 2 | 3 | 6 | 8 | 2 | 12 | 4 | 4 | 8 | 12 | 5 | 12 | 5 | 6 | 6 | 12 | 4 | 6 | 2 |
| **Cycle 5. PT + I (B_A_) Training** | | | | | | | | | | | | | | | | | | | | | | | | | | | | | | |
| **Pure Tact** | 13. | Yes | 12 | 12 | 12 | 12 | 12 | 12 | 12 | 12 | 12 | 12 | 12 | 12 | 12 | 12 | 12 | 12 | 12 | 12 | 12 | 12 | 12 | 12 | 12 | 12 | 12 | 12 | 12 | 12 |
| **Pure Tact** | 14. | Yes | 12 | 12 | 12 | 12 | 12 | 12 | 12 | 12 | 12 | 12 | 12 | 12 | 12 | 12 | 12 | 12 | 12 | 12 | 12 | 12 | 12 | 12 | 12 | 12 | 12 | 12 | 12 | 12 |
| **Intraberval** | 15. | Yes | 12 | 12 | 12 | 12 | 12 | 12 | 12 | 12 | 12 | 12 | 13 | 12 | 12 | 12 | 12 | 12 | 12 | 12 | 12 | 12 | 12 | 12 | 12 | 12 | 12 | 12 | 12 | 12 |
| **Intraberval** | 16. | Yes | 12 | 12 | 12 | 12 | 12 | 12 | 12 | 12 | 12 | 12 | 13 | 12 | 12 | 12 | 12 | 12 | 12 | 12 | 12 | 12 | 12 | 12 | 12 | 12 | 12 | 12 | 12 | 12 |
| **Cycle 6. IT Post-test (A_B2_)** | | | | | | | | | | | | | | | | | | | | | | | | | | | | | | |
| **Impure Tact** | 17. | No | 12 | 12 | 2 | 3 | 12 | 11 | 12 | 12 | 5 | 12 | 10 | 12 | 12 | 2 | 12 | 12 | 12 | 3 | 12 | 12 | 12 | 12 | 11 | 12 | 3 | 1 | 5 | 6 |
